# Testing the amount of nicotinamide mononucleotide and urolithin A as compared to the label claim

**DOI:** 10.1007/s11357-024-01257-2

**Published:** 2024-06-27

**Authors:** E Sandalova, H Li, L Guan, SD Raj, TG Lim, E Tian, BK Kennedy, AB Maier

**Affiliations:** 1 Healthy Longevity Translational Research Programme, Yong Loo Lin School of Medicine, National University of Singapore, Singapore, 117456 Singapore; 2 Centre for Healthy Longevity, National University Health System (NUHS), Singapore, Singapore; 3 Centre of Innovation, for Complementary Health Product (COI-CHP) Temasek Polytechnic, Singapore, Singapore; 4 Departments of Biochemistry and Physiology, Yong Loo Lin School of Medicine, National University of Singapore (NUS), Singapore, Singapore; 5 Department of Human Movement Sciences, @AgeAmsterdam, Faculty of Behavioural and Movement Sciences, Vrije Universiteit Amsterdam, Amsterdam Movement Sciences, Amsterdam, Netherlands

**Keywords:** Supplements, Geroprotector, Quality, Nicotinamide mononucleotide, Urolithin A

## Abstract

Healthy Longevity Medicine aims to optimize health by targeting aging processes across the lifespan. Addressing accelerated aging involves adaptation of lifestyle and the use of geroprotective drugs and supplements, including nutritional supplements and bioactive compounds. The Food and Drug Administration, under the Dietary Supplement Health and Education Act, categorizes bioactive compounds and medicinal products as dietary supplements. While numerous companies sell ingredients that can be deemed geroprotectors, there's limited oversight in their quality control. Governmental safety authorities only verify the presence of prohibited compounds, not the accuracy of ingredients listed on labels.

Here, Nicotinamide mononucleotide and Urolithin A supplements, easily accessible online or in pharmacies, were tested for their active ingredient content. Results showed a significant deviation from the labeled amounts, ranging from + 28.6% to -100%. This indicates a considerable disparity in the quality of geroprotective supplements.

To address this variability, collaboration between and within societies representing healthcare professionals, industry and regulatory bodies is imperative to ensure the quality of geroprotective supplements.

**Supplementary Information:**

The online version contains supplementary material available at 10.1007/s11357-024-01257-2.

## Introduction

Geroscience aims to understand the mechanisms of aging that contribute to the development of age-related diseases that limit lifespan [1]. Geroprotectors are compounds that interfere with aging mechanisms and protect against aging [2]. Many of those are categorized as health supplements, food additive or nutraceuticals rather than drugs [2–8] and thus, are regulated by the Food Drug Administration (FDA) under the Dietary Supplement Health and Education Act of 1994 (DSHEA). Before marketing products, manufacturers and distributors of dietary supplements must ensure product safety and labeling in accordance with the requirements of the Federal Food, Drug, and Cosmetic Act as amended by DSHEA [9, 10]. They should also comply with the Generally Recognized As Safe (GRAS) rule, stating that the substance added to food is considered safe by experts under the conditions of its intended use and must not claim to treat, cure or prevent disease [3, 11]. DSHEA classified herbals and other medicinal products as dietary supplements without emphasizing any requirements for quality of these products. Thus, there are no uniform standards for supplements, nor is there consistent reinforcement of compliance [12]. Investigation of the concentration of melatonin obtained from drug stores in Canada showed that the content of melatonin is highly variable, and some supplements also included serotonin, Gamma-aminobutyric acid (GABA) and various plant extracts [13]. Content of melatonin in melatonin gummies sold in the US was also variable from non-detectable to + 347% of labeled quantity [14]. Eleven out of 25 vitamin D3 and K2 supplements showed 95% alignment with the claimed amount [15]. Many supplements include combinations of various components without assessing the potential interaction or food-matrix effect, which might reduce the concentration or mode of action of active compounds [16] and the bioavailability of its ingredients [17].

Nicotinamide Mononucleotide (NMN) has gained significant attention as a potential geroprotective compound. NMN has been reported to be safe and well-tolerated in healthy adults [18–20] and shown to increase (1) the levels of Nicotinamide Adenine Dinucleotide (NAD +) in human blood [20, 21], (2) insulin sensitivity in pre-diabetic women [22], (3) aerobic capacity in amateur runners [23], and (4) sleep quality in older adults [24]. It is an isometric compound with a complex structure, and it is difficult and costly to produce [25]. In addition, many supplement producers add other ingredients to NMN; it is unclear if matrix effects [26] can hinder the recovery of NMN. A commercial study conducted by Chromadex reported that 14 out of 22 tested NMN products contained less than 1% of NMN, and some had no detectable NMN [27].

Urolithin A (UA) is a microbial metabolite of ellagic acid and related polyphenols derived from foods such as pomegranates, strawberries, raspberries, walnuts, and others [28]. Approximately 40% of healthy individuals possess the necessary microbial species for converting ellagic acid into UA [29, 30]. UA supplementation has been shown to be safe and to increase UA concentration in blood in middle-aged adults [31, 32]. In addition, UA improves muscle endurance, decreases plasma levels of acylcarnitines, ceramides and C-reactive protein [33], and improves muscle performance [31]. Common supplements include UA or pomegranate extract with defined or undefined content of ellagic acid.

Here, eighteen NMN and five UA supplements underwent high-performance liquid chromatography coupled to triple quadrupole mass spectrometry testing to determine the amount of active ingredients present, which were then compared to the quantities stated on their respective labels.

## Methods

### NMN and UA supplements

**Fig. 1:**
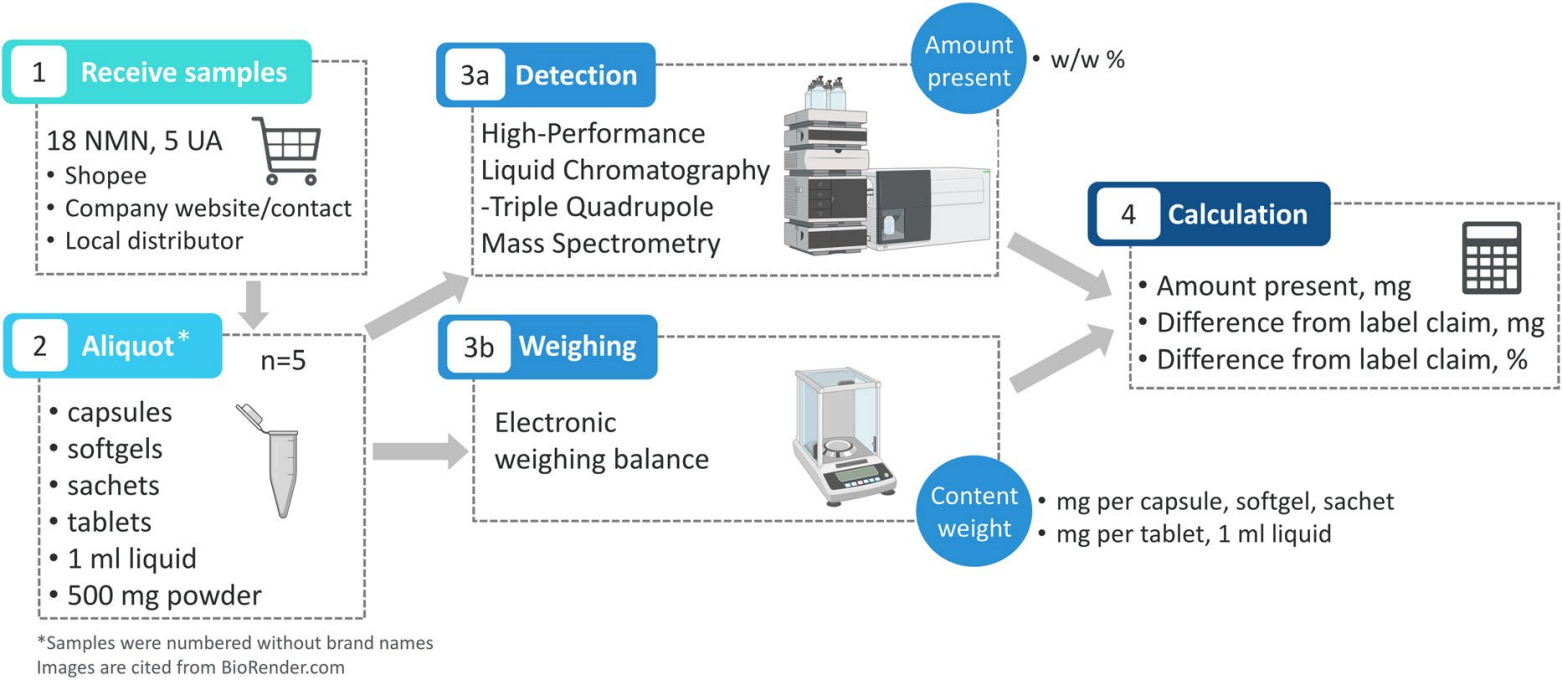
Schematic representation of methods. NMN and UA supplements were purchased (1), aliquoted and labelled NMN#1-NMN#18 for NMN and UA#1-#5 for UA (2) and sent to laboratory for HPLC-QqQ-MS five aliquots/sample (3a). In parallel each dose of supplement was weighed as a whole and content without the shell/packaging (3b). Finally, the amount present and the difference from adjusted label claim was calculated (4)

**Fig. 2:**
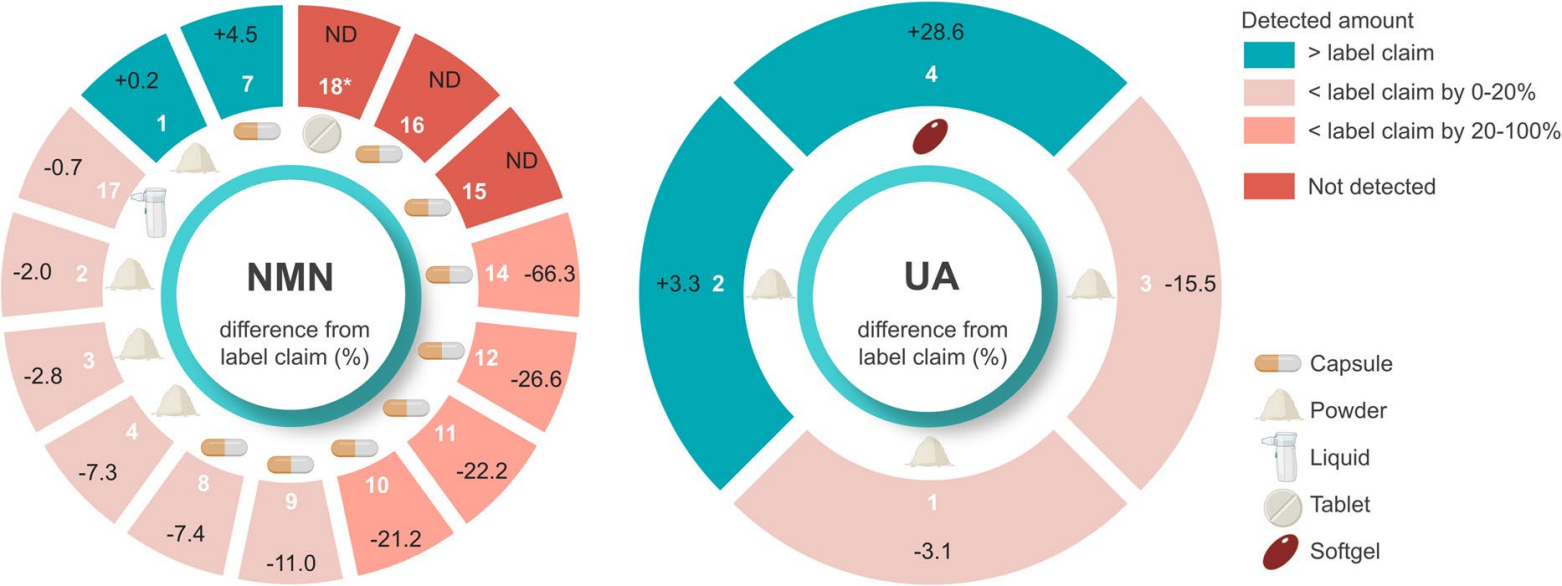
The difference from adjusted label claim of non-liposomal NMN (A) (NMN#1 to NMN#4, NMN#7–12 and NMN#14–18) and UA (B) (UA#1–4). UA#5 was not included as it did not claim any Urolithin A on the label. Adjusted label claim amount was subtracted from the detected amount of NMN or UA and expressed in percent

Eighteen NMN (15 non-liposomal, 3 liposomal) and five UA supplements were purchased online (Fig. 1, (1)), in pharmacies or acquired directly from manufacturers and stored according to manufacturer’s instructions. Information on the reported purity and the cost of each supplement was extracted. The cost was recalculated for 1 g of NMN or UA. To blind the measurements and analyses, supplements were aliquoted and randomly numbered by a researcher not involved in the study (NMN#1–18 and UA#1–5) (Fig. 1, (2)). Supplements provided as powder were aliquoted into vials of 500 mg, and capsules or tablets were separated into one capsule or tablet per vial. The reported amount of active ingredient (NMN or UA), here referred to as label claim, was recorded for each supplement, together with manufacturing and expiry dates. NMN powder under product code NMN#1 was used to produce Liposomal NMN powder and capsules for products coded NMN#5 and NMN#13.

NMN supplements were tested in two batches. The first batch included ten NMN supplements (codes NMN#1–10, measured 02/05/2023) and the second batch eight NMN supplements together with the supplement out of the first batch with the highest and lowest measured deviation from the label claim (NMN#11–20, measured 24/09/2023). Urolithin A supplements were analyzed on 22/2/2023 (UA#1–5).

### High performance liquid Chromatography-Triple quadrupole mass spectrometry

The content of NMN was measured using High Performance Liquid Chromatography-Triple Quadrupole Mass Spectrometry (HPLC-QqQ-MS, Agilent Technologies, USA) (Fig. 1, (3a)) which was developed and validated for this purpose. An Agilent 6495C triple quadrupole LC/MS and Agilent 1290 Infinity HPLC System comprising of a G4220A binary pump, a G4226A autosampler, and a G1330B thermostat (Agilent Technologies, USA) were used for quantitative analysis. Standards were set as NMN T4721, Afirmus Biosource, Singapore and UA SML1791, Sigma-Aldrich Pte Ltd, USA. Prior to the analysis, each capsule was cut open, tablet crushed, and powder weighed, 50 mg of each NMN supplement and 100 mg of UA supplement was reconstituted in 50 mL of Ultrapure (Milli-Q Integral 5 system, Merck, Germany) water for NMN and 10 mL of Dimethyl sulfoxide (DMSO) for UA. The liquid NMN supplement was diluted in Ultrapure water. After centrifugation, all samples were filtered through 0.45um membranes into High-performance liquid chromatography (HPLC) vials. Agilent Poroshell 120 EC-C18 (3.0 × 15 mm, 2.7 μm) (Agilent Technologies, USA) column was used with column temperature 25 °C. Mobile phase flow rate was 0.4 mL/min, with injection volume was 2 μL, mass-to-charge ratio (m/z) of precursor ion was 335.07 and m/z of ion was 122.9 and 97.1. The collision energy was 9 and 5 eV respectively. The stop time was 11 min and post time was 4 min. Ion source parameters were the following: gas temperature 250 °C, gas flow 16 L/min, nebulizer 35 psi, sheath gas temperature 300 ºC, sheath gas flow 11 L/min.

The recovery of the extraction was calculated by dividing the concentration from equally mixed samples (NMN samples mix NMN#1–10 and mix NMN#11–20 and UA samples mix UA#1–5) that were spiked before extraction and samples spiked after the extraction. The recovery fell within the acceptable range of 80%-120%. Precision was determined by calculating the coefficient of variation (CV) of replicates within one sample run. Acceptable result was defined as CV < 10% [34, 35]. Accuracy was defined as the relative deviation in the calculated value of a standard from that of its true value, expressed as relative error (RE). Acceptable RE was defined as lower than 10% [34, 35]. Matrix effect was calculated through peak area of NMN in equally mixed sample matrix from NMN sample mix NMN#1–10, mix NMN#11–20 and UA sample mix UA#1–5 over peak area of the same amount of NMN in blank methanol. The acceptable range shall be 95%-105% [34, 35]. A series of standard solutions with varying NMN/UA concentrations were prepared via serial dilution of the stock reference standard solution. The standard solutions were then injected consecutively into the LC–MS, and their peak areas (y) were plotted against their concentrations (x, ng/mL) to establish a standard curve with regression equation given by y = ax + b. The % content and SD of NMN/UA in each sample were calculated using the equation [(y – b)/a] /weight of sample (mg) × 100% (i.e. present mean, % ± present SD, %). The mean of the present mean (%) and present SD (%) of repeatedly tested products from two batches was used as the result of the repeated measured NMN supplements (for product codes NMN#3 and NMN#11).

### Calculations of difference of claim label amount and measured amount of NMN and UA

The claimed NMN amount on the label was recorded (mg) and adjusted by purity (%) if reported (adjusted label claim amount, mg = label claim amount × purity claim). As for powder format, 1000 mg was used as label claim amount. For the sachet format, each sachet was weighed. Separately, the net weight (powdered contents) was weighed 5 times without the external packaging. Each capsule or tablet was weighed 5 times as the whole capsule or tablet and as content of the capsule without the shell. Liquid was pumped twice into an Eppendorf tube and weighed (Fig. 1, (3b)). The mean weight and standard deviation (SD) were calculated (content mean, mg ± content SD, mg). The NMN/UA present amount (mg) and SD (mg) was calculated as present mean (%) × content mean (mg) or content SD (mg). Difference from adjusted label claim amount (mg) was calculated as NMN present amount (mg)—adjusted label claim amount (mg). Difference from adjusted label claim (%) was calculated as difference from adjusted label claim amount (mg) / adjusted label claim amount (mg) × 100% (Fig. 1, (4)).

## Results

### Supplements

The list of NMN and UA supplements tested are given in Table 1 and Supplementary Table 1. Five out of 18 products tested were in powder format; eleven were capsules, one was a tablet, and one was a liquid format (99.8–99% purity when reported). The cost for consumers of 1 g of NMN supplement ranged from 1.1 to 17.5 Euro, for 1 g of UA/Pomegranate extract from 5.8 to 65.4 Euro.

**Table 1:** Content of non-liposomal and liposomal NMN and Urolithin A supplements

Product Code, #	Format	Label claim amount, mg/capsule/sachet or softgel or g or ml	Purity claim, %	Adjusted label claim amount, mg/capsule or sachet or softgel or g or ml	Amount present, mean ± SD, w/w%	Content weight, mean ± SD, mg	Amount present, mean ± SD, mg	Difference from adjusted label claim, mg	Difference from adjusted label claim, %
Nicotinamide Mononucleotide									
NMN #1	powder	1000	> 99	990	99.2 ± 11.8	1000	992.2	2.2	0.2
NMN #2 ^^	powder	1000	99.8	998	97.8 ± 7.2	1000	978.2	-19.8	-2.0
NMN #3	powder	1000	> 99	990	96.2 ± 5.6	1000	961.9	-28.2	-2.8
NMN #4 ^^	powder	1000	n.r	1000	92.7 ± 7.9	1000	927.4	-72.6	-7.3
NMN #5 liposomal	powder	1000	> 99	990	13.5 ± 2.2	1000	135.4	-854.6	-86.3
NMN #6 liposomal	capsule	250	n.r	250	44.4 ± 2.3	626.7 ± 17.6	278.0 ± 7.8	28.0	11.2
NMN #7	capsule	500	n.r	500	91.6 ± 8.7	570.1 ± 81.7	522.4 ± 74.9	22.4	4.5
NMN #8	capsule	500	98	490	98.3 ± 7.4	461.6 ± 71.8	453.6 ± 70.6	-36.4	-7.4
NMN #9	capsule	500	n.r	500	98.2 ± 12.1	453.1 ± 49.9	444.8 ± 49.0	-55.2	-11.0
NMN #10	capsule	450	n.r	450	58.8 ± 4.5	603.8 ± 35.5	354.8 ± 20.9	-95.2	-21.2
NMN #11	capsule	160	n.r	160	29.4 ± 1.8	422.8 ± 37.2	124.5 ± 10.9	-35.5	-22.2
NMN #12	capsule	250	99	247.5	63.0 ± 9.4	288.3 ± 15.0	181.6 ± 9.4	-65.9	-26.6
NMN #13 liposomal	capsule	125	> 99	123.8	13.1 ± 1.4	628.3 ± 18.3	82.4 ± 2.4	-41.4	-33.4
NMN #14	capsule	500	n.r	500	48.3 ± 3.1	348.4 ± 39.3	168.4 ± 19.0	-331.6	-66.3
NMN #15	capsule	500	n.r	500	n.q	473.8 ± 2.6	0	-500	-100
NMN #16	capsule	250	n.r	250	n.q	410.8 ± 17.1	0	-250	-100
NMN #17	liquid	50	n.r	50	4.7 ± 0.2	1061.4 ± 12.1	49.7 ± 0.6	-0.3	-0.7
NMN #18	tablet	0	n.r	0	n.q	899.4 ± 7.2	n.a	n.a	n.a
Urolithin A									
UA#1	powder	1000	98	980	95.0 ± 0.7	1000	950.0	-30.0	-3.1
UA#2	powder	3000	16.67	500.1	15.1 ± 0.4	3421.2 ± 160.4	516.6 ± 24.2	16.6	3.3
UA#3	powder	1000	99	990	83.7 ± 2.2	1000	837.0	-153.0	-15.5
UA#4	softgel	250	n.r	250	35.7 ± 3.1	900.4 ± 5.4*	321.5 ± 1.9	71.5	28.6
UA#5	capsule	0	n.r	0	n.q	403.9 ± 11.7	n.a	n.a	n.a

NMN: Nicotinamide mononucleotide. n.r.: Not reported; n.a.: Not applicable; n.q.: Not quantifiable

Adjusted label claim: consider reported purity

^*^Based on entire capsule weight

^ lactose, indigestable maltodextrin, L-arginine, fruit and vegetable extract (corn), soluble fiber, agaricus blazei murill, solid phase culture), asparagus, polygunatum odoratum, eucommia leaves, emlical seeds, figs, green beans, turmeric, eleuthero, cassia, plantain, chamomile flower, chamomile leaf, bellflower, black fungus, cat's claw wine extract, caggabe, guava, guava leaf, wolfberry, wolfberry leaf, xiongshi, grapes, grapefruit, burdock, coriander, perilla leaves, perilla seeds, ginger, horsetail, star fruit, elderberry, celery, dandelion root, pepper, houlluynia, red dates, carrots, honeysuckle, garlic, pineapple, parsley, passion fruit, coix seed, banana, green papaya, sweet pepper, loquat, loquat leaf, antler sea salt, spinach, maca, kiwi fuit, Wenzhou tangerine, melon, bean sprouts, jute seven, mugwort leaf, dragon palace flower, polugonum root, apple, rooibois tea, ganoderma lucidum fruiting bodies, ganoderma lucidum mycelium, lemon, saffron, lotus mist, soybean, yam, onion, licorice, kelp, pine leaves, kumquat, persimon leaves, mulberry leaves, mulberry fruit, plum, pear, black beans, shiitake mushrooms, matcha, lotus root, mangosteen, honeysuckle, apricote pit, red algae powder, cauliflower powder, spirulina powder, black maca powder, zinc, gluconate, poyvinylpyrrolidone, magnesium stearate, avocado extract, nicotinamide (0.9 mg NE is reported for nicotinic acid and nicotinamide)

^^ NMN supplier for supplement companies

The characteristics of the supplements are presented in Supplementary Table 2. Most NMN and UA supplements were white powders, whether in capsules, in a sachet, softgel or tablet form. One NMN supplement was a transparent liquid. UA#5 product was a pomegranate extract, specified to contain ellagic acid.

### Date

Five out of 18 NMN supplements had manufacturing dates reported (NMN#17, NMN#16, NMN#7, NMN#6 liposomal NMN#15) and 9 out of 18 reported the expiry date (NMN#2, NMN#3, NMN#11, NMN#18, NMN#15, NMN#4, NMN#10, NMN#14, NMN#16, NMN#1) (Supplementary Table 1). One out of five UA supplements reported manufacturing date (UA#2) and two reported expiry date (UA#3, UA#5).

### Weight of supplements

The weight of the NMN supplement’s whole capsules ranged from 0.39 g (NMN#12) to 0.73 g (NMN#13 liposomal capsules), whereas the weight of the content of the capsules was 0.29 g (NMN#12) to 0.63 g (NMN#13 liposomal capsules). The content of the capsule (total weight – shell weight) represented 73.9% (NMN#14) to 86.6% (NMN#6 liposomal) of the total capsule weight. The weight of UA supplements ranged from 0.9 ± 0.054 g (UA#4 softgel contents) and 0.51 ± 0.012 g, content weight 0.4 ± 0.012 g (UA#5 capsule) (Table 1).

### NMN content of supplements

The measured NMN content, w/w% (weight of detected NMN/weight of total content %), ranged from non-detectable (NMN#15, NMN#16, NMN#18) to 99.2% (NMN#1) NMN of total sample weight (Table 1). The difference from adjusted label claim ranged from -100% (NMN#15, NMN#16, NMN#18) (Table 1, Fig. 2) to + 11.2% (NMN#6) (Table 1).

### UA content of supplements

The measured UA content, w/w% (weight of detected UA/weight of total sample %), ranged from non-detectable (UA#5) to 95% (UA#1) UA of total sample weight (Table 1). The difference from adjusted label claim ranged from -15.5% (UA#3) to 28.6% (UA#4 softgel) (Table 1, Fig. 2). UA#5 was not included in the calculation of the difference as it did not claim any Urolithin A on the label.

### Matrix effect

The matrix effect refers to the recovery of the ingredient of interest from the mixture of ingredients. The matrix effect for both NMN and UA supplements was within the acceptable range of 95%-105% (Supplementary Table 3).

## Discussion

NMN and UA health supplements, sold online and/or in pharmacies, contain variable amounts of NMN and UA differing from the label. Most of the supplements contained lower amounts compared to label quantities; two of 15 non-liposomal NMN and two of four UA products had higher amounts.

The data suggest that supplements showing lower detected amounts, the active ingredient is degrading, or lower amounts added, while in supplements with higher-than-labeled amounts, larger quantities are intentionally added to counteract a breakdown. These larger quantities are referred to as overages and are common in supplement industry [36]. Since the matrix effect was not observed in tested supplements, it is unlikely that the recovery of NMN or UA from the mixture was a problem. However, it is not excluded that adding additives could compromise stability and efficacy, since reported clinical trials only evaluate the effect of single ingredient. A previous report from ChromaDex found similar results, where 22 brands were tested, and 14 samples were below 1% of NMN claimed amount and three samples had no NMN detected. There was no overlap in the brands tested in this report and Chromadex report, except for one product (NMN#15), which yielded similar results [27].

For some ingredients it could be difficult to ensure that the product is homogeneously dissolved, thus, the equal distribution between the capsules could pose a challenge. Because UA has low solubility [37], it's unclear if manufacturers employ any techniques to enhance its solubility for the softgel. If UA is used in suspension, the unequal distribution between capsules is possible.

Liposomes have emerged as one of the most promising tools for drug delivery systems [38]. Supplements have also utilized liposomal systems for improving bioavailability and bioaccessibility [39]. Here, three liposomal NMN supplements were tested together with non-liposomal products and showed varied NMN content. This applied method was not developed and validated for testing specifically the liposomal products, the accuracy of the results for liposomal products cannot be confirmed to be equivalent to that of non-liposomal NMN products. It was expected that liposomes would be dismantled by the sonication step of the sample preparation, however, it has not been tested whether the liposomes were indeed destroyed and released all the NMN contained. The liposomal techniques employed, like LIPO-DRY in two of the liposomal products, pose challenges in effectively breaking down the liposomes, as suggested by the manufacturer (personal communication).

The strength of the study is that very stringent methods for testing NMN and UA were used. Methods were developed and validated for each experiment, including testing the recovery, precision, accuracy, and matrix effect. High-performance liquid chromatography coupled to triple quadrupole mass spectrometry offers quantification of analytes with high sensitivity and specificity, especially applicable for lower concentrations [40]. Each dose of the supplement, capsule, tablet, softgel or sachet was weighed and the actual NMN was calculated. This step has shown that the weight of the capsule content was already lower than the amount on the label.

Geroprotective supplements have great potential to optimize health of aging individuals. Therefore, quality control is of the utmost importance. All manufacturers were informed about their individual results and eleven manufacturers of NMN products and all of UA products have responded to the results, thus, the dialogue striving for a better quality of the geroprotective supplements was initiated.

## Conclusion

The amount of active ingredient of NMN and UA indicated on the label poorly corresponds to the detected amount. Therefore, reinforcement of regulations for geroprotective supplements is needed.

### Supplementary Information

Below is the link to the electronic supplementary material.

Supplementary file1 (DOCX 30 KB)

## Data Availability

Data will be made available on request.
