# Supplementary material for: Testing the amount of nicotinamide mononucleotide and urolithin A as compared to the label claim
Source: GeroScience. 2024 Jun 27;46(5):5075–83. doi: 10.1007/s11357-024-01257-2 (PMC11335992; doi:10.1007/s11357-024-01257-2)
Supplement: Supplementary file 1 — Supplementary file1 (DOCX 30 KB) [file 11357_2024_1257_MOESM1_ESM.docx]

**Supplementary Table 1**. Description of NMN and Urolithin A supplements.

| Product Code | | Details | | Format, N per bottle | Amount/ serving, mg | | Price/ 1g, Euro | Manufacturing date | Expiry date | Additional information |
| --- | --- | --- | --- | --- | --- | --- | --- | --- | --- | --- |
| Nicotinamide mononucleotide (NMN) | | | | | |  |  |  |  |  |
| NMN#1 | | ß-NMN | | powder | 500 | | n.a. | n.r. | 11/2024 | Lot: 2209C-23 |
| NMN#2 | | ß-NMN | | powder | n.r. | | n.a. | n.r. | n.r. | CAS No: 1094-61-7. Batch No: 5206-2209010 |
| NMN#3 | | ß-NMN | | powder | up to 1000 | | 2.5 | n.r. | 01/2024 | Batch: XJY01220116 |
| NMN#4 | | NMN | | powder | n.r. | | n.a. | n.r. | n.r. | n.r. |
| NMN#5 liposomal | | Liposomal NMN, high-quality Lipo-Dry liposome | | powder | n.r. | | n.a. | n.r. | n.r. | n.r. |
| NMN#6 liposomal | | ß-NMN (250 mg), vegetable cellulose (capsule), sunflower lecithin, rice flour, microcrystalline cellulose | | capsules 90 | 500 | | 1.4 | 07/2023 | n.r. | BN: 07255441 |
| NMN#7 | | ß-NMN (500 mg), Hydroxypropyl methylcellulose (HPMC) | | capsules 60 | 500 | | 2.5 | 04/04/2023 | n.r. | Vers: 02.22.2023. Lot: 230471 |
| NMN#8 | | ß-NMN (500 mg), capsule shell (Hydroxypropyl Methylcellulose) | | capsules 30 | 500 | | 3.1 | n.r. | n.r. | n.r. |
| NMN#9 | | Uthever NMN (500 mg), cellulose | | capsules 60 | 1000 | | 1.5 | n.r. | 11/2024 | IB 65150 |
| NMN#10 | | ß-NMN (450 mg), Vit D (D3 Cholecalciferol from Lichen, 10 mcg), Trans-Resveratrol (98% Poligonum cuspidatum root extract, 50 mg), Olea25® (25% hydroxytyrosol, olive leaf plant extract, 40 mg), Ergothioneine (2 mg), Cellulose (capsule), Nu-GLOW (organic) rice concentrate | | capsules 60 | 900 | | 3 | n.r. | 09/2025 | Lot: 0222050 |
| NMN#11 | | ß-NMN (160 mg), Reservatrol (200 mg), Ginseng peptide (40 mg) | | capsules 30 | 160 | | 17.5 | n.r. | 06/2025 | BN: G220501 |
| NMN#12 | | ß-NMN (250 mg), vegetable cellulose (capsule), microcrystalline cellulose | | capsules 30 | 250 | | 5.5 | n.r. | n.r. | n.r. |
| NMN#13 liposomal | | Liposomal NMN with high quality sunflower lecithin (non-GMO), Lipo-Dry liposome | | capsules | 125 | | n.a. | n.r. | n.r. | n.r. |
| NMN#14 | | NMN (500 mg) | | capsules 30 | 500 | | 2.7 | n.r. | 06/2024 | Batch No: 062022 |
| NMN#15 | | ß-NMN (500 mg), cellulose (veggie capsule) | | capsules 60 | 500 | | 1.1 | 02/01/2022 | 01/01/2025 | Lot: GDZ-14-13 |
| NMN#16 | | NMN (250 mg), Quercetin (250 mg), Trans-resveratrol (200mg), Astragalus (100 mg), L-Theanine (100 mg), Curcumin (100 mg), Collagen (50 mg), CoQ10 (50 mg), Crocin (50 mg), Epigallocatechin Gallate (50 mg), Fisetin (50 mg), silicon dioxide, magnesium stearate, rice flour, vegetable cellulose | | capsules 60 | 500 | | 7.3 | 17/05/2022 | 16/05/2024 | Lot: 220501 |
| NMN#17 | | ß-NMN (50 mg), Trimethylglycine (50 mg) (as betaine), water, glycerin, ethanol, phospholipids (from purified sunflower seed lecithin), tocofersolan, natural mixed tocopherols | | liquid 50 ml | 50/ml | | 1.4 | 10/2022 | n.r. | Product code: QSNMN-1, 15199.06. NMN2205/1 |
| NMN#18 | | plant extracts, nicotinamide listed as one of the ingredients # | | tablets 30 | n.a. | | n.a. | n.r. | 26/09/2024 | n.r. |
|  |  | |  | | |  |  |  |  |  |
| UA#1 | | 98% pure | | powder | n.r. | | 65.37 | n.r. | n.r. | n.r. |
| UA#2 | | Blueberry powder, Raspberry powder, Rice Fiber, Pomegranate Extract, Natural flavor | | powder | 500 | | 11.53 | 07/11/2022 | n.r. | Lot: 072211D-02 |
| UA#3 | | 99% pure | | powder | n.r. | | 13.50 | n.r. | 02/2024 | Batch: XJY16220202 |
| UA#4 | | MCT (medium-chain triglyceride) oil, Sunflower Oil, Gelatin, Glycerol, Purified Water, Glycerol Monostearate, Red Iron Oxide Color | | softgel, 30 | 500 | | 5.77 | n.r. | n.r. | n.r. |
| UA#5 | | Pomegranate fruit extract (Punica Granatum) (40% Ellagic Acid = 100 mg) | | capsule, 50 | 0 | | NA | n.r. | 06/2023 | Lot: 1653EV1984 |

n.r.: Not reported; n.a.: Not applicable; n.q.: Not quantifiable.

**Supplementary Table 2.** Physical description of supplements

| Product Code | Sample Description: NMN |
| --- | --- |
| NMN#1 | White powder |
| NMN#2 | White powder |
| NMN#3 | White powder |
| NMN#4 | White powder |
| NMN#5 liposomal | White powder |
| NMN#6 liposomal | Non-transparent capsule, content is white powder |
| NMN#7 | Non-transparent capsule, content is white powder |
| NMN#8 | Transparent capsule, content is white powder |
| NMN#9 | Transparent capsule, content is white powder |
| NMN#10 | Transparent Capsule, content is white powder |
| NMN#11 | Non-transparent capsule, content is white powder |
| NMN#12 | Non-transparent capsule, content is white powder |
| NMN#13 liposomal | Transparent capsule, content is white powder |
| NMN#14 | White powder |
| NMN#15 | Non-transparent capsule, content is white powder |
| NMN#16 | Half blue half white capsule with white powder |
| NMN#17 | Liquid |
| NMN#18 | Capsule, content is light greenish powder |
|  | Sample Description: UA |
| UA#1 | Granular powder, light grey in colour |
| UA#2 | Granular powder, light grey in colour |
| UA#3 | Granular powder, light brown in colour |
| UA#4 | Red pill containing pale yellowish viscous cream |
| UA#5 | Translucent pill containing dark grey granular powder |

**Supplementary Table 3.** Matrix Effect of NMN (NMN#1-10 and NMN#11-18) and UA Supplements

| Product code | Matrix effect (%) | RSD (%) |
| --- | --- | --- |
| NMN#1-10 | 104.7 $\pm$ 0.03 | 2.9 |
| NMN#11-18 | 96.74 ± 7.55 | 7.8 |
| UA#1-5 | 101.4 $\pm$ 0.3 | 0.32 |

RSD, relative standard deviation
